# Diagnostic Complexities in Congenital Tuberculosis: Insights From Preterm Twins Conceived via In Vitro Fertilization and a Literature Review

**DOI:** 10.1155/crdi/8016755

**Published:** 2026-09-25

**Authors:** Mei-Juan Zhou, Jian Yu, Feng-Xia Huang, Ning Wang, Qing Wang

**Affiliations:** ^1^ Department of Neonatology, Children’s Hospital of Soochow University, NO92 Zhongnan Street, Industrial Park, Suzhou 215025, Jiangsu, China, sdfey.cn

**Keywords:** CTB, FGTB, immunological discordance, in vitro fertilization, tuberculosis screening

## Abstract

Congenital tuberculosis (CTB) poses significant diagnostic challenges, particularly in preterm infants. This case report describes the discordant clinical presentation of CTB in dizygotic twins conceived via in vitro fertilization (IVF) and born at 31^+6^ weeks’ gestation. Both twins presented with nonspecific symptoms (fever and respiratory distress) on postnatal day (PND) 31 and 25, respectively, but followed distinct clinical courses: Twin 1 developed disseminated disease with facial palsy and central nervous system (CNS) involvement, whereas Twin 2 presented with a cervical mass and bone destruction. Diagnosis was markedly delayed, with confirmation on PND 99 and 98, respectively, and initially mistaken for sepsis or histiocytosis. Definitive diagnosis was achieved through next‐generation sequencing (NGS) and Xpert Mycobacterium tuberculosis/Rifampicin (Xpert MTB/RIF) assays. The twins were treated with a 14‐month antitubercular regimen including linezolid. Their asymptomatic mother was subsequently diagnosed with latent genital TB—a likely contributor to her primary infertility—and received standard therapy with isoniazid (INH), rifampicin (RIF), pyrazinamide (PZA), and ethambutol (EMB). At follow‐up (chronological age 2 years), both twins showed adequate catch‐up growth and normal visual function. This case illustrates that CTB can manifest as phenotypically diverse and disseminated disease in preterm IVF twins following reactivation of maternal latent infection. It also provides a clinical paradigm linking female genital tuberculosis (FGTB) pathogenesis—mediated by Th1/Th17‐driven endometrial damage—to infertility, IVF‐associated immunosuppressive reactivation, and congenital transmission. These findings highlight the potential value of considering latent TB screening in women undergoing infertility evaluation, particularly before IVF, to prevent severe maternal and neonatal outcomes. However, given that these observations are derived from a single case report, larger studies are warranted to establish definitive screening recommendations.

## 1. Introduction

Tuberculosis (TB) continues to pose a major global public health challenge, with an estimated 10.8 million new cases reported in the WHO Global Tuberculosis Report 2024. Children and adolescents account for 12% of this burden [1]. Congenital tuberculosis (CTB), a manifestation within this pediatric demographic, is exceptionally rare yet severe and often fatal. Its true population‐based incidence remains undefined, representing a significant gap in epidemiological knowledge. The historical prognosis of CTB underscores its severity, with early literature describing near‐universal mortality in the absence of treatment. A comprehensive 2011 review of 170 global cases (1946–2009) documented persistently high case fatality rates, which showed only a modest decline from 52.6% in the pre‐1994 period to 33.9% thereafter [2]. Contemporary evidence corroborates this evolving yet still grave outlook, alongside persistent diagnostic challenges. A 2019 systematic review of 92 CTB cases in China reported an initial misdiagnosis rate of 59.8% and an overall mortality of 43.5%. Crucially, mortality dropped to 15.4% among infants who received a confirmed diagnosis followed by prompt and appropriate antitubercular therapy [3]. The most encouraging contemporary data originate from a 2025 multicenter cohort study by the Paediatric Tuberculosis Network European Trials Group, comprising 27 CTB cases [4]. It demonstrated that active, standardized management can reduce the case fatality rate to as low as 3.7%. This estimate requires cautious interpretation, however, as it likely excludes neonates who die prior to establishment of a definitive diagnosis. Thus, CTB presents a profound clinical paradox: extreme rarity coexists with a high risk of diagnostic error and a stark disparity in outcomes between promptly treated and undiagnosed cases. This dichotomy constitutes a direct and substantial threat to survival. To address this gap, we present a twin case analysis with a dual aim: first, to highlight common diagnostic pitfalls in concurrent CTB; and second, to use this unique model to demonstrate the molecular basis of divergent clinical phenotypes in dizygotic twins, as well as to elucidate pathogenic mechanisms linking maternal latent female genital tuberculosis (FGTB) to infertility.

## 2. Case Report

We present a case of CTB in preterm dizygotic male twins conceived via in vitro fertilization (IVF), detailing their clinical manifestations, diagnostic evaluations, management strategies, and factors predisposing to initial misdiagnosis.

### 2.1. First‐Born Twin

**FIGURE 1:**
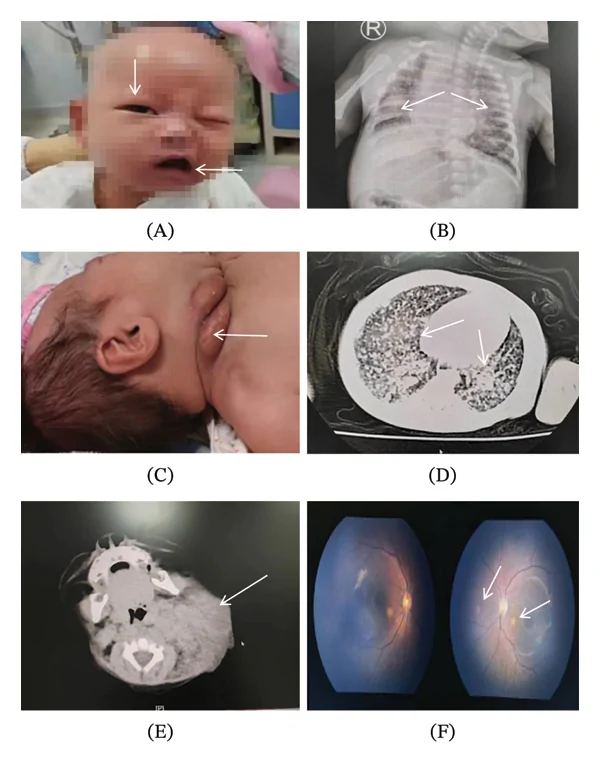
(A) First‐born twin, facial palsy. (B) First‐born twin (PND95), chest X‐ray image exhibiting pneumonia. (C) Second‐born twin, cervical mass. (D) Second‐born twin, pulmonary CT showing miliary changes (PND95). (E) Second‐born twin, CT scan of the neck showed a large cystic mass. (F) Second‐born twin, two yellow lesions in the posterior fundus of the left eye.

A male preterm infant was delivered by cesarean section at 31^+6^ weeks of gestation, with a birth weight of 1760 g. Upon admission to the neonatal intensive care unit (NICU), continuous positive airway pressure support, parenteral nutrition, and empirical antimicrobial therapy were promptly initiated, with subsequent initial clinical improvement. On postnatal day (PND) 31, the infant developed recurrent fever and dyspnea, and chest radiography revealed mildly increased interstitial lung markings. Due to progressive respiratory decompensation, he was transferred to a tertiary care center on PND 35, where he was diagnosed with neonatal respiratory failure, pneumonia, anemia, retinopathy of prematurity, and nephrolithiasis. Despite broad‐spectrum antimicrobial therapy (meropenem, vancomycin, and fluconazole) and oxygen support, his condition deteriorated, necessitating invasive mechanical ventilation and the addition of linezolid. Despite worsening chest radiographic findings, the infant was discharged against medical advice on PND 62. He was readmitted to our NICU on PND 95 due to failure to thrive, poor feeding, and the emergence of new symptoms, including unilateral left facial palsy (Figure 1A), cough, hepatosplenomegaly, lymphadenopathy, abdominal distension, opisthotonus and hypertonia, as well as chorioretinal lesions. Abdominal ultrasound demonstrated hepatomegaly. The mesentery was thickened and hyperechoic with multiple hypoechoic nodules, findings highly consistent with tuberculous infiltration. Chest X‐ray demonstrated bilateral pulmonary infiltrates (Figure 1B), and empirical therapy with latamoxef and amoxicillin‐sulbactam was initiated. The infant had not received the Bacille Calmette–Guérin (BCG) vaccine prior to the onset of illness. The cerebrospinal fluid (CSF) and blood bacterial and fungal cultures were all negative.

Comprehensive diagnostic evaluations confirmed mycobacterium tuberculosis (MTB) infection: next‐generation sequencing (NGS) of blood and sputum samples detected MTB DNA, the Xpert MTB/RIF assay was positive for MTB nucleic acid without RIF resistance, and both the interferon‐gamma release assay (IGRA) and T‐SPOT. TB tests were positive (Table 1). CSF analysis and abdominal ultrasonography supported a diagnosis of disseminated disease, which was definitively confirmed as disseminated TB on PND 99 [5] (Table 2).

**TABLE 1:** Auxiliary examination.

	Specimen	First‐born twin	Second‐born twin
Xpert MTB/RIF	Sputum	+/−	+/−
	CSF	NO	—

IGRA	Blood	+	+

TSPOT	Blood	+	+

NGS (sequence reads)	Blood	MTB + (3)	MTB + (3)
	Sputum	MTB + (625)	—
	CSF	NO	—
	Cervical mass pus	NO	MTB + (53975)

TB antibody		—	—

CSF WBC		12 × 10ˆ^6^/L	46 × 10^6^/L

CSF Lac		0.9 mmol/L	1.11 mmol/L

CSF protein		1168 mg/L	1602 mg/L

CSF glucose		3.4 mmol/L	2.4 mmol/L

CSF chlorinum		110.9 mmol/L	114 mmol/L

Tissue biopsy		NO	Cervical mass: inflammatory granuloma with fibrinoid necrosis, no LCH

*Note:* +: positive; −: negative; NO: no related item.

Abbreviation: WBC, white blood cell.

**TABLE 2:** Diagnosis of CTB according to the Cantwell criteria.

	First‐born twin	Second‐born twin
The infant proved tuberculous lesions	Yes	Yes
Lesions in the first week of life	No	No
Hepatic primary complex	No	No
Maternal genital tract infection	Yes	Yes
Exclusion of postnatal infection	Yes	Yes

Following resolution of a left common femoral vein thrombosis treated with urokinase, anti‐tubercular therapy was initiated on PND 101 with isoniazid (INH), rifampicin (RIF), pyrazinamide (PZA), and linezolid (LZD) after obtaining informed consent. The patient was transferred to a specialized infectious disease unit on PND 103.

### 2.2. Follow‐Up and Outcomes

After 8 months of antitubercular treatment (at 1 year of chronological age), CSF parameters returned to normal and facial palsy showed improvement. He completed a 14‐month anti‐TB treatment regimen, with sustained symptomatic improvement during a subsequent 7‐month follow‐up period. At a corrected age of 10 months (1 year of chronological age), Griffiths Scales results were as follows: Domain A (11.5 months), Domain B (8.5 months), Domain C (8.5 months), Domain D (9 months), and Domain E (9 months). At a corrected age of 1 year and 10 months (2 years of chronological age), his height was 83 cm (15th percentile, P15), weight was 10.45 kg (P15), and head circumference was 48.3 cm (50th percentile, P50) [6].

### 2.3. Second‐Born Twin

The second‐born twin, weighing 1600 g, was admitted to the NICU immediately after delivery. Initial clinical management was consistent with that administered to his twin brother. Recurrent fever and dyspnea developed on PND 25—earlier than they did in his twin brother. Due to failure to improve symptoms by PND 35, the patient was transferred to a higher level secondary hospital. A chest X‐ray performed on PND 25 revealed slightly increased pulmonary markings. Upon transfer to the secondary hospital, the patient received anti‐infective therapy comprising meropenem, vancomycin, and fluconazole. Nevertheless, gradual enlargement of a cervical mass was noted (Figure 1C). Pathological examination of the cervical mass yielded inconclusive results, with negative results of both acid‐fast bacilli (AFB) staining and fungal cultures. The patient was diagnosed as a preterm low birth‐weight infant complicated by Langerhans cell histiocytosis (LCH), infectious fever, cervical lymphadenopathy, bone destruction, severe anemia, otitis externa, and external auditory canal eczema. The patient was discharged against medical advice on PND 77, despite persistent low‐grade fever. Due to worsening clinical manifestations including fever, dyspnea, cervical mass, hepatosplenomegaly, bone destruction, lymphadenopathy, otitis externa, anemia, and growth retardation, he was readmitted to our Pediatric Intensive Care Unit on PND 93. NGS of peripheral blood and cervical mass pus specimens both detected MTB DNA, whereas direct sputum samples and CSF yielded negative results. The Xpert MTB/RIF assay confirmed MTB nucleic acid positivity in sputum with no RIF resistance gene detected, while CSF tested negative for MTB nucleic acid. Additionally, both the IGRA and T‐SPOT. TB tests were positive (Table 1). CSF examination was performed (Table 1). The CSF and blood bacterial and fungal cultures were all negative. Pulmonary CT revealed miliary lesions (Figure 1D), while cervical CT demonstrated a large cystic mass accompanied by adjacent bone destruction (Figure 1E). Abdominal CT was unremarkable, whereas abdominal ultrasound demonstrated hepatomegaly. Histopathological examination of the neck lesion revealed inflammatory granuloma and inflammatory fibrinoid necrosis (Table 1). On PND 98, merely 3 days after hospitalization, a diagnosis of disseminated systemic TB was established [5] (Table 2), and antitubercular treatment with INH, RIF, PZA, and LZD was promptly initiated. His symptoms gradually improved after the initiation of antitubercular therapy, with fever resolving completely 10 days later. After 8 months of antitubercular therapy (at 1 year of chronological age), CSF parameters returned to normal. He subsequently completed a 14‐month treatment course, and his symptoms continued to improve during a 7‐month follow‐up period thereafter. At a corrected age of 10 months (chronological age 1 year), Griffiths Scales assessments were as follows: A (9 months), B (8.5 months), C (9 months), D (7 months), and E (9 months). At a corrected age of 1 year and 10 months (chronological age 2 years), his height was 82.6 cm (near the 15th percentile), weight 10.95 kg (P5–P15), and head circumference 49.1 cm (P50–P85) [6]. The patient had not received the BCG vaccine prior to the onset of illness.

The twin patients were monitored in accordance with a standardized protocol throughout antitubercular treatment. Hepatic and renal function, along with complete blood counts, were assessed monthly for the first 6 months, after which the frequency was reduced to trimonthly until treatment completion. However, only mild and transient hepatic impairment was observed, which did not necessitate therapy interruption. Regarding ophthalmological surveillance, visual acuity was assessed at 2, 6, and 12 months after treatment initiation, as well as at 4 and 7 months (patient age: 2 years) after treatment cessation. All results of vision screening were normal.

### 2.4. The Mother

The mother was asymptomatic with a history of primary infertility and no known history of TB until her twins were diagnosed with CTB. Following her infants’ TB diagnosis, additional diagnostic workup revealed acute hematogenous disseminated pulmonary TB (Supporting Video A) and intracranial tuberculoma on magnetic resonance imaging (MRI) (Supporting Video B). She presented with a persistently nonhealing cesarean incision; upon confirmation of TB, Xpert MTB/RIF assay of incision exudate tested positive for MTB nucleic acid, with no evidence of rifampicin resistance. Xpert MTB/RIF assays of CSF and sputum were negative. The patient was initiated on a four‐drug antitubercular regimen consisting of INH, RIF, PZA, and ethambutol (EMB). Following treatment, her cesarean incision healed completely. After completion of treatment, subsequent chest CT indicated near‐complete resolution of miliary pulmonary lesions, and brain MRI demonstrated significant regression of intracranial tuberculomas (Supporting Video A and B). She was discharged with a final diagnosis of acute hematogenous disseminated pulmonary TB, intracranial tuberculoma, and pelvic and genital TB.

## 3. Discussion

The clinical courses of the twins illustrate several key points regarding diagnosis and pathogenesis. Due to the closed management of the NICU and visitation restrictions during the COVID‐19 pandemic in China, the twins were admitted to the NICU immediately after birth without any maternal contact or parental care. They remained separated from their mother from birth until PND 62 and 77, respectively, and their clinical symptoms developed during this separation period. According to the standardized diagnostic criteria for distinguishing CTB from postnatal TB [5], postnatal transmission was therefore excluded in both cases. CTB is transmitted via two principal routes: hematogenous placental dissemination and amniotic fluid aspiration [4, 7, 8]. Hematogenous spread, considered the major pathway for intrauterine infection, occurs when MTB invades the placenta, establishes granulomatous lesions, and subsequently enters the fetal circulation via the umbilical vein. The organisms first seed the fetal liver, forming a primary hepatic complex, and then disseminate systemically. In contrast, amniotic fluid aspiration results from the contamination of amniotic fluid, often due to maternal genital tract TB. The fetus aspirates infected fluid in utero or during delivery, leading to primary infection of the respiratory or gastrointestinal mucosa. From a pathophysiological perspective, placental granulomas compromise the integrity of the placental barrier, thereby facilitating MTB transplacental translocation into the fetal bloodstream. Furthermore, preterm infants exhibit immature skin [9]and mucosal barriers as well as underdeveloped innate and adaptive immune defenses [10], which collectively increase their susceptibility to MTB infection. In this case, the mother presented with a persistently nonhealing cesarean incision, which was not initially attributed to TB. However, after she was diagnosed with multisystem TB, Xpert MTB/RIF assay of the incision exudate returned positive for MTB nucleic acid, confirming the diagnosis of pelvic genital TB. Together with the twins’ complete isolation from the mother prior to symptom onset, which effectively excludes postnatal transmission, this provides strong clinical evidence for the diagnosis of CTB. However, in the absence of placental histopathological examination, we cannot definitively distinguish between hematogenous placental dissemination and amniotic fluid aspiration as the specific route of intrauterine transmission.

A constellation of nonspecific clinical and radiographic features substantially delayed diagnosis. Although the age at onset in the twins was consistent with previous reports (typically 2–3 weeks, maximum 154 days [3, 11]), their age at diagnosis exceeded 3 months. CTB typically presents with nonspecific manifestations, including fever, respiratory distress, and hepatomegaly [2–4]. Abdominal ultrasound demonstrated hepatomegaly in both twins, but no definite hepatic granulomas or caseous necrosis were identified. Although hepatic granulomas or fibrinoid necrosis remain the diagnostic gold standard, their invasiveness, combined with the growing diagnostic utility of noninvasive modalities (NGS, IGRA, and T‐SPOT) in recent years, has limited the role of liver biopsy in current CTB‐related literature. Most recent studies have instead focused on abdominal imaging, which is frequently unremarkable; when abnormalities are present, they are primarily hepatosplenomegaly or hypoechoic nodules, observed in approximately 23.1%–40% of cases [12, 13]. This is likely due to the inherent limitations of imaging compared with pathological examination. The Paediatric Tuberculosis Network European Trials Group previously reported that fewer than one‐quarter of CTB patients undergoing abdominal imaging showed evidence of primary hepatic complexes or caseating granulomas, prompting the group to propose less restrictive modified diagnostic criteria [4]. Furthermore, percutaneous liver biopsy was considered clinically and ethically challenging in this young cohort. Procedural risks in infants, together with the fact that biopsy would not have altered management or prognosis in light of positive findings from less invasive modalities, argued against the procedure. Furthermore, parental consent was not obtained. Therefore, liver biopsy was not performed. In addition to the aforementioned symptoms, the twins presented with left facial palsy, opisthotonos, hypertonia, chorioretinal lesions, and a cervical mass. MTB infiltration into the CNS can trigger a severe inflammatory response. Facial palsy might be attributed to direct compression or infiltration of the facial nerve. Involvement of deep motor control centers (e.g., basal ganglia, pyramidal tracts) reduced inhibitory signals, leading to hypertonia characterized by sustained limb muscle over‐contraction. Extension of inflammation to the spinal cord or specific brainstem regions induced intense extensor spasms, manifesting as opisthotonos. CNS involvement was indicated by facial nerve palsy in the first‐born twin and abnormal CSF findings in the second‐born twin. Both manifestations resolved completely following antitubercular therapy, despite prior failure of broad‐spectrum antibiotic treatment. Definitive microbiological confirmation of CNS TB was not achieved in either twin (first‐born twin: CSF NGS/Xpert not performed; second‐born twin: CSF NGS negative), rendering the diagnosis clinical. The complexity of presenting symptoms obscured differentiation between CTB and conditions including pneumonia, septicemia, purulent meningitis, and pulmonary complications of prematurity [4, 14]. Key differential diagnoses were systematically excluded as outlined below. First, bacterial sepsis and pneumonia were ruled out by persistently negative blood and CSF cultures, together with a lack of clinical response to broad‐spectrum antimicrobial therapy (including meropenem, vancomycin, and fluconazole). Second, fungal infection was excluded by negative fungal cultures and no clinical improvement with antifungal treatment. Third, LCH—initially suspected in the second‐born twin due to cervical mass and bone destruction—was ruled out by histopathological examination of the cervical lesion, which demonstrated inflammatory granuloma and fibrinoid necrosis in the absence of pathognomonic Birbeck granules or CD1a/Langerin‐positive cells; subsequent detection of MTB DNA by NGS further confirmed a tuberculous etiology. Fourth, purulent meningitis was excluded by negative CSF bacterial cultures, absence of other pathogens on CSF NGS, and ultimate identification of MTB as the causative agent. Collectively, these findings confirmed CTB as the definitive diagnosis over other initial considerations. Despite the established diagnosis, it is important to note that both chest imaging and routine laboratory tests showed nonspecific abnormalities. While chest radiography and CT demonstrated severe pulmonary inflammation, no rales were detected on auscultation [3, 4]. Laboratory abnormalities in CTB—including anemia, thrombocytopenia, elevated leukocyte count, C‐reactive protein, and procalcitonin—closely mimic those observed in bacterial and fungal infections [15]. Notably, these twins were both diagnosed with CTB but displayed distinct clinical phenotypes, in contrast to a previous report in which only one twin was affected while the co‐twin remained healthy [16].

To account for the phenotypic discordance observed between the twins, we propose that dichorionic placentation in this IVF‐conceived dizygotic twin pair likely resulted in differential initial MTB inocula and distinct fetal immune milieus. Given that neonates typically exhibit relative Th1 hyporesponsiveness and a predisposition toward immune tolerance [17, 18], we further postulate that in Twin 1, an insufficient Th1 response coupled with excessive type I interferon production undermined effective granuloma formation. This, in turn, promoted hematogenous spread to the central nervous system (CNS), clinically manifesting as facial nerve palsy and meningeal irritation signs [19, 20]. In Twin 2, a comparatively robust Th17‐predominant immune response likely exacerbated peripheral inflammation, thereby promoting cervical mass formation and bone destruction [21–25]. Nevertheless, these immunological pathways were not directly assessed in our cases and remain inferred from the literature. Genetic modifiers—including human leukocyte antigen and Toll‐like receptor polymorphisms—may have further contributed to the phenotypic divergence observed between the co‐twins [26, 27].

In this case, the mother presented with a persistently nonhealing cesarean incision, which was not initially attributed to TB. However, after she was diagnosed with multisystem TB, Xpert MTB/RIF assay of the incision exudate returned positive for MTB nucleic acid, confirming the diagnosis of pelvic genital TB. Given the absence of any TB‐related history or symptoms in the asymptomatic mother described in this case, no targeted TB screening was performed prior to embryo transfer. Furthermore, the possibility of TB infection was not considered during the initial evaluation of the twins’ complex clinical presentations. Therefore, the case also highlights the diagnostic challenge posed by asymptomatic maternal FGTB. This diagnostic delay is likely attributable to several factors: the low prenatal detection rate of TB [14, 28], the frequently asymptomatic nature of FGTB, and insufficient clinical awareness of this condition. The absence of systemic symptoms can lead to the underrecognition of infertility secondary to FGTB [15], which is particularly concerning given that infertility may be its sole clinical manifestation—indeed, 40%–80% of women with FGTB experience infertility [29]. To contextualize these challenges, a brief review of FGTB pathophysiology is warranted. Accounting for 15%–20% of extrapulmonary TB cases [30], FGTB has an estimated prevalence of approximately 1% in the United States [31]. FGTB typically originates from the hematogenous, lymphatic, or contiguous spread of MTB from a primary site of infection [30]. Hematogenous dissemination most commonly seeds the fallopian tubes (95%–100%), followed by the endometrium (50%–60%), ovaries (20%–30%), cervix (5%–15%), myometrium (2.5%), and vagina/vulva (1%) [30]. Molecularly, MTB infection induces a Th1/Th17 immune skew, marked by elevated IFN‐γ, TNF‐α, and IL‐17 [22, 32]. This cytokine milieu drives caseating granuloma formation and fibrosis [22], culminating in structural sequelae such as tubal occlusion and intrauterine adhesions [30]. Furthermore, FGTB downregulates key receptivity markers (cadherin‐1, mucin 1, peripheral lymph node addressin, and integrin αvβ3) and inhibits the leukemia inhibitory factor–STAT3 signaling axis, thereby reducing vascular endothelial growth factor transcription and ultimately impairing endometrial receptivity [30, 33]. Additionally, MTB exerts an anti‐gonadotrophic effect in FGTB, impairing progesterone and human chorionic gonadotropin production. In patients with FGTB, elevated luteinizing hormone and follicle‐stimulating hormone levels, accompanied by markedly reduced inhibin, indicate impaired ovarian function and reserve [30]. These mechanisms collectively underpin the fertility impairment frequently observed in FGTB. The clinical significance of these mechanisms becomes especially pronounced when infertile women with FGTB conceive via IVF. During pregnancy, cell‐mediated immunity is compromised during pregnancy due to a relative shift toward T‐helper 2 (Th2)‐biased immunity [34]. Routine progesterone and glucocorticoid supplementation following IVF further contributes to immunosuppression: progesterone exerts a dose‐dependent effect on Th1/Th2 responses, reducing T‐cell proliferation and host immunity [35], while glucocorticoids inherently suppress immunity. Moreover, elevated estrogen levels during pregnancy promote the proliferation of MTB [36]. Collectively, pregnancy‐associated immunosuppression increases the risk of latent tuberculosis infection (LTBI) reactivation, leading to low successful childbirth rates and high risks of CTB, abortion, maternal mortality, and complications that endanger both maternal and fetal lives [8, 37, 38]. Notably, most mothers of infants with IVF‐associated CTB did not undergo routine TB screening during IVF preparation [39]. Therefore, TB screening prior to IVF—especially for asymptomatic FGTB—may be warranted before IVF treatment initiation [39]. Tal et al. have proposed that at‐risk women undergoing infertility evaluation could be screened for LTBI using risk stratification questionnaires and the QuantiFERON‐TB Gold assay; those with positive results may undergo endometrial biopsy for further diagnostic workup [31]. Nevertheless, the optimal screening strategy remains to be determined in future prospective studies.

The gold standard for diagnosing TB remains mycobacterial culture for MTB. However, culture is often time‐consuming and may exhibit low sensitivity. Diagnosis in infants is particularly challenging. Consequently, molecular methods such as Xpert MTB/RIF and NGS are increasingly utilized in clinical practice [38]. Traditional techniques, including acid‐fast staining, IGRA, T‐SPOT. TB, and tuberculin skin tests (TST), provide supporting diagnostic information. In the present case, NGS enabled rapid and definitive diagnosis, with no rifampin‐resistance‐associated mutations identified. Although preterm infants generally exhibit impaired immune function and lower IGRA sensitivity compared to older children [40], our patients nevertheless mounted a cellular immune response to tuberculous infection, as evidenced by positive IGRA and T‐SPOT. TB results. Reported positivity rates of IGRA and T‐SPOT. TB in culture‐confirmed CTB are 65.5% and 84.2%, respectively [13, 41]. These positive results provide molecular and cellular evidence supporting the CTB diagnosis. Regarding treatment, linezolid exhibits excellent penetration into key TB disease sites, including pulmonary cavities [42], CSF [43, 44], and bone [45, 46]. Given the disseminated nature of the patient’s disease, involving both the brain and bone, linezolid [12, 47] was added to the first‐line regimen (INH, RIF, and PZA), with treatment duration adjusted based on clinical response and lesion severity [48]. The twins completed a 14‐month antituberculosis treatment course, demonstrating continuous clinical improvement throughout an additional 7‐month follow‐up period. Treatment was administered in an isolated private ward under strict infection control protocols. Two months after discharge, all attending physicians and nurses who had close contact with the twins tested negative for TB by both T‐SPOT. TB and TST. According to previous reports, INH prophylaxis is commonly administered to high‐risk neonates exposed to active TB cases during hospitalization to prevent nosocomial transmission [49]. Rifampin or levofloxacin has also been used effectively as window prophylaxis or for the treatment of LTBI in newborns, with no cases of active TB reported during the 1‐year follow‐up period [50].

## 4. Conclusions

CTB remains a diagnostic challenge associated with potentially severe outcomes, particularly in preterm infants. This report of dichorionic dizygotic twins conceived via IVF underscores the remarkable phenotypic heterogeneity of CTB. The observed diagnostic delay illustrates how its nonspecific clinical presentation often mimics more common neonatal conditions, such as sepsis. A key insight from this case is the association between latent FGTB and primary infertility. We posit a clinical cascade in which undiagnosed FGTB contributed to infertility, and subsequent IVF‐associated hormonal therapies likely precipitated reactivation and vertical transmission. These observations necessitate a high index of suspicion for CTB in preterm infants with unexplained, protracted illness. More importantly, they suggest that latent TB screening may warrant consideration as part of the pre‐IVF evaluation of women undergoing fertility treatment, though larger studies are needed to confirm this approach. As demonstrated by the twins’ catch‐up growth and positive neurodevelopmental trajectory, favorable outcomes can be achieved through timely diagnosis and individualized antitubercular therapy. Ultimately, this case reinforces that CTB should not be viewed merely as a neonatal disease, but rather as a potential consequence of silent maternal infection. A proactive diagnostic approach in both the infertility workup and neonatal care settings is therefore crucial to mitigating severe infant morbidity and mortality. It must be emphasized, however, that these conclusions are derived from a single case report and should be interpreted with caution until further evidence from larger cohort studies becomes available.

## 5. Limitations and Future Directions

Despite the valuable insights provided, this study has several limitations that should be acknowledged. First, the single‐center retrospective design may introduce selection bias, potentially limiting the generalizability of the results to the broader population of infertile women undergoing IVF. Furthermore, the lack of histological evaluation of maternal placental tissue restricts our ability to explore potential placental‐related mechanisms underlying the observed outcomes. To address these limitations, future prospective multicenter studies are warranted to minimize selection bias and improve external validity. Incorporating systematic TB screening in infertile women receiving IVF, along with detailed placental histological assessment, would help elucidate the role of TB in IVF outcomes and strengthen the evidence base for clinical guidelines in reproductive medicine.

## Funding

No funding was received for this manuscript.

## Ethics Statement

This study was approved by the Institutional Review Board of our hospital (approval no. 2026CS033). We confirm that written informed consent for the use of all clinical data, including videos and images, was obtained from the parents of the twins prior to manuscript preparation and publication.

## Conflicts of Interest

The authors declare no conflicts of interest.

## Supporting Information

Additional supporting information can be found online in the Supporting Information section.

## Supporting information


**Supporting Information** (1) Video A shows serial chest CT images of the mother obtained before and after treatment, demonstrating significant radiological improvement with near‐complete resolution of the miliary pattern. (2) Video B shows serial cranial MRI images of the mother before and after treatment, demonstrating significant radiological improvement with marked regression of intracranial tuberculomas. The following raw data are also provided as supporting files for reference: Blood NGS results of the first‐born twin were positive for MTB, with 3 sequence reads. Sputum NGS results of the first‐born twin were positive for MTB, with 625 sequence reads. Blood NGS results of the second‐born twin were positive for MTB, with 3 sequence reads. NGS results of pus from the cervical mass in the second‐born twin were positive for MTB, with 53,975 sequence reads. Histopathological examination of the cervical mass in the second‐born twin revealed inflammatory granuloma and inflammatory fibrinoid necrosis.

## Data Availability

The data that support the findings of this study are available in the supporting information of this article.
