# Supplementary material for: Diagnostic Complexities in Congenital Tuberculosis: Insights From Preterm Twins Conceived via In Vitro Fertilization and a Literature Review
Source: Case Rep Infect Dis. 2026 Sep 25;2026:8016755. doi: 10.1155/crdi/8016755 (PMC13614344; doi:10.1155/crdi/8016755)
Supplement: Supplementary file 1 [file CRDI-2026-8016755-s001.zip › Blood NGS results of the first-born twin.pdf]

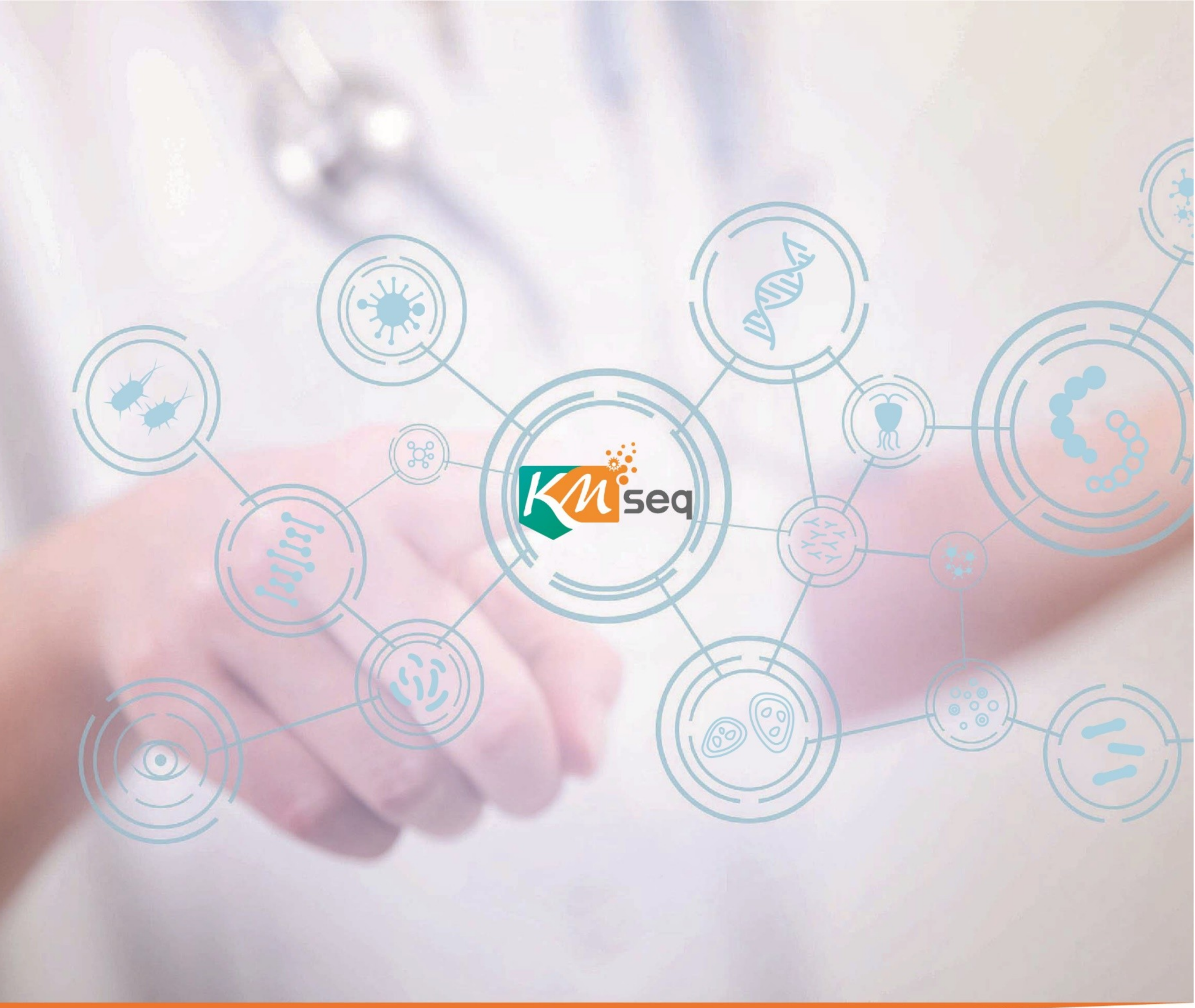

DNA-病原微生物宏基因组检测报告单

姓名:	[redacted]	性别:	男	年龄:	3月6天
医院:	苏州大学附属儿童医院-检验科				
样本类型:	血液				
报告日期:	2022-06-04				

金域客服

声明：本检测为科研检测，且仅对该条码号对应的标本负责，如果对结果有疑义，请在收到报告单后3个工作日内与我们联系，多谢合作！

金域医学
KingMed Diagnostics

CS002M82HHHQZ4V

金域医学 KingMed Diagnostics

DNA-病原微生物宏基因组检测报告单

电话：4001-111-120　　网址：www.kingmed.com.cn

受检者信息

姓名	[redacted]	性别	男	年龄	3月6天
送检医院	苏州大学附属儿童医院-检验科				
送检科室	新生儿24区	送检医生	汪清	住院号	[redacted]
标本类型	血液	标本条码	0641061596	实验号	DM01D4943
采样日期	2022-06-02	接收日期	2022-06-03	报告日期	2022-06-04

临床信息

临床诊断	重点关注病原：结核杆菌				
主诉	咳嗽，发热1周				
体温	-	白细胞计数	15.37×10^9/L	中性粒细胞数目/比例	69.7%
淋巴细胞数目/比例	15.9%	单核细胞数目/比例	12.8%	嗜酸粒细胞数目/比例	-
红细胞沉降率	-	血小板计数	344×10^9/L	血红蛋白	79g/L
降钙素原	0.27ng/mL	C反应蛋白	91.22mg/L	PCR	Xpert：阳性
G试验	-	GM试验	-	隐球菌荚膜多糖抗原	-
镜检/培养	-		病原相关血清学检测	-	
病理	-				
影像	两肺炎症，肠郁张				
前期抗感染方案及疗程	拉氧头孢+阿莫西林钠舒巴坦钠，2日				

DNA-病原微生物宏基因组检测结果	阳性

该标本检出结核分枝杆菌（序列数:3），检出疑似定植菌和/或背景微生物，未检出耐药基因。

请结合患者临床表现和其它辅助诊断结果综合判断。

1.特殊病原体列表（分枝杆菌、支原体、衣原体、立克次体、螺旋体等）

属				复合群/种		
类型	名称	序列数	相对丰度	名称	序列数	覆盖度
G+	分枝杆菌属 *Mycobacterium*	17	0.25%	结核分枝杆菌 *Mycobacterium tuberculosis*	3	0.01%

2.细菌列表

属				复合群/种		
类型	名称	序列数	相对丰度	名称	序列数	覆盖度
未检出						

3.真菌列表

属				复合群/种		
类型	名称	序列数	相对丰度	名称	序列数	覆盖度
未检出						

4. DNA 病毒列表

属				种/型/亚型		
类型	名称	序列数	相对丰度	名称	序列数	覆盖度
未检出						

5.寄生虫列表

种名	序列数	相对丰度
未检出		

6.疑似定植和/或背景微生物列表

表示通过生信分析和人工审核后无法明确判断的可疑微生物，包含可能引起感染的未达到过滤条件的真正病原体，采样和操作过程中由试剂、耗材或环境引入的条件致病菌，以及人体常见的定植微生物，请结合患者临床表现及其它辅助检查，综合判断。

属			复合群/种	
类型	名称	序列数	名称	序列数
ssDNA	甲型细环病毒属<br>*Alphatorquevirus*	2	细环病毒 29 型<br>*Torque teno virus 29*	2

7.耐药基因列表

耐药基因	对应物种	序列数	覆盖度	对应耐药抗微生物药物
未检出				

注释：

1.本检测基于 CARD 数据库，从 2900 多种耐药基因中挑选出较为常见的 63 种，包括 *mecA/B/C* 、 *vanA/B/C* 、 *ermA/B/C* 、 *blaOXA* 、 *blaCTX-M*等，未包含由核苷酸位点突变引发的耐药。

2.阴性结果解读注意事项：由于耐药基因相对较小，且受到样本中人源核酸的影响，应用微生物宏基因组检测分析耐药基因性能不稳定，检出灵敏度受微生物检出序列数影响较大。

3.阳性结果解读注意事项：耐药基因检出与实际耐药表型并不完全一致，因此检出耐药基因仅供临床参考，请临床综合患者情况谨慎使用检测结果。

病原体解释说明（附阳性病原基因组覆盖图）

结核分枝杆菌(*Mycobacterium tuberculosis*)该菌是革兰阳性菌，隶属于分枝杆菌属，是结核分枝杆菌复合群的一员，是结核病的病原体，可产生索状因子和硫脂，人类对其有较高的易感性，最易受损的器官是肺，能侵犯全身各器官，可引起肺结核、脑膜炎等。

*Mycobacterium tuberculosis*基因组覆盖图(覆盖率：<0.01%)

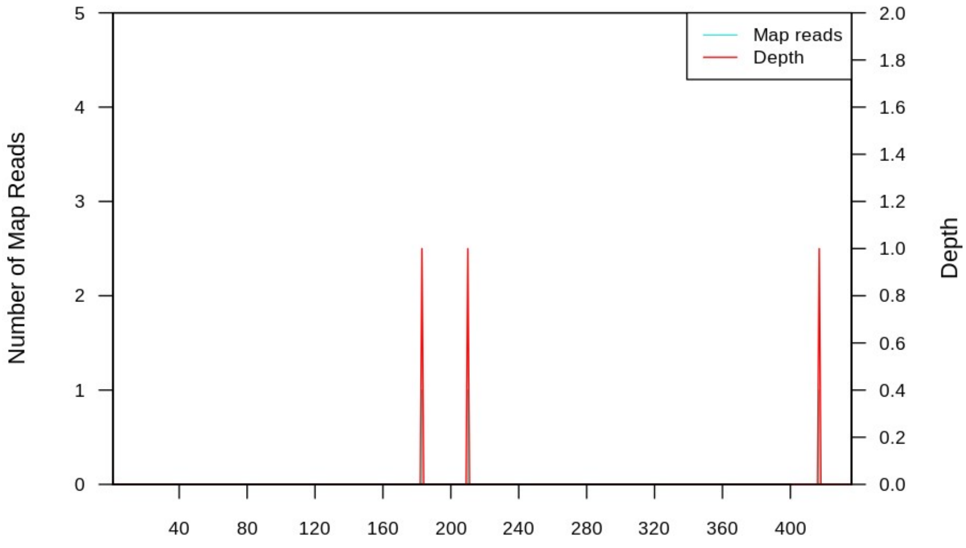

Nucleotide Position(X10K) Genome size: 4,359,659

细环病毒29型(*Torque teno virus 29*)该病毒的宿主并不只局限于人，在除人之外的灵长动物（黑猩猩、猕猴、胥、夜猴）及树鼩、猫、狗中都检测到该病毒，同时也广泛存在于周遭环境中，如地表水等。细环病毒分布广泛，并且任何携带的个体都有可能成为潜在的传染源。目前对于细环病毒的传播途径有多种说法，其中血液传播被认为是其主要传播途径之一，同时其可以借助唾液滴实现呼吸道传播。细环病毒感染者可以借助粪、食物、水等媒介实现肠道传播。另外，由于细环病毒在全球有较高的流行率，胎盘传播、性传播也被认为是其传播途径之一。其他一些观点认为接触感染者的皮肤、毛发也有可能被感染。细环病毒在健康个体中有较高的检出率，无明显易感倾向。地中海贫血患者、长期血液透析患者、凝血因子治疗的血友病患者及静脉药瘾患者的血清或血液中细环病毒检出率会更高。有研究认为人细环病毒与包括肝炎、癌症、发热、血液系统疾病在内的疾病有关，但尚无直接证据表明临床疾病与该病毒的直接关系。

名词解释

1、检出序列数：将总序列数标准化至 20M reads 后，能够高质量比对到该病原体的序列数目，其多少与标本中病原体本身的载量以及人源序列数占比有关。检出序列数越高，通常表示该病原体的载量越高。

2、相对丰度：将病原体按照细菌、真菌、病毒和寄生虫进行分类，相对丰度是该病原体相应分类中序列数的相对比例。相对丰度越高，通常表示该病原体在标本中的占比越高。

3、覆盖度：检出序列覆盖到的基因组长度占物种基因组总长度的比例。覆盖度越高，通常表示检出该病原体的真实度越高。

阳性结果解读

重点关注病原菌列表，建议结合患者临床表现及其它辅助检查进行判断。

疑似阳性结果解读

1、重点关注病原菌列表，建议结合患者临床表现及其它辅助检测方法进行验证；

2、定植微生物以及人群中常见携带的 EBV、CMV、细环病毒等病原体，需综合患者临床表现及其它辅助检查进行判断。

疑似阴性结果解读

重点关注补充微生物列表，请结合临床以及其它检测结果进行验证。

阴性结果解读

1、送检标本中病原微生物的含量低于最低检测下限，或取材部位非主要感染部位；

2、送检标本非感染标本，请医生结合患者临床表现及其它辅助检查综合判断，排除感染。

参考文献

1. Michael M J , Binnicker M J , Sheldon C , et al. A Guide to Utilization of the Microbiology Laboratory for Diagnosis of Infectious Diseases: 2018 Update by the Infectious Diseases Society of America and the American Society for Microbiology[J]. Clinical Infectious Diseases, 2018, 67(6):813-816.

2. Chiu C Y , Miller S A . Clinical metagenomics[J]. Nature Reviews Genetics, 2019, 20(6): 341-355.

3. Byrd A L , Belkaid Y , Segre J A . The human skin microbiome[J]. Nature Reviews Microbiology, 2018, 16(3).

4. He T , Kaplan S , Kamboj M , et al. Laboratory Diagnosis of Central Nervous System Infection[J]. Current Infectious Disease Reports, 2016, 18(11):35.

5. Miller S , Naccache S N , Samayoa E , et al. Laboratory Validation of a Clinical Metagenomic Sequencing Assay for Pathogen Detection in Cerebrospinal Fluid[J]. Genome Research, 2019, 29(5): 831-842.

6. Huffnagle G B , Dickson R P , Lukacs N W . The Respiratory Tract Microbiome and Lung Inflammation: A Two-way Street[J]. Mucosal Immunology, 2017, 10(2):299-306.

常见病原微生物检测列表（项目类型：各类病原体测定）

细菌

金黄色葡萄球菌	血链球菌	肺炎克雷伯菌	艰难梭菌	布鲁氏菌	鲍曼不动杆菌
表皮葡萄球菌	牛链球菌	催产克雷伯菌	脆弱类杆菌	鼠疫耶尔森菌	卡他莫拉菌
溶血葡萄球菌	屎肠球菌	奇异变形杆菌	具核梭杆菌	小肠结肠炎耶尔森菌	嗜水气单孢菌
人葡萄球菌	粪肠球菌	普通变形杆菌	小韦荣球菌	炭疽芽孢杆菌	豚鼠气单胞菌
路登葡萄球菌	脑膜炎奈瑟菌	阴沟肠杆菌	痤疮丙酸杆菌	蜡样芽胞杆菌	单核细胞李斯特菌
沃氏葡萄球菌	淋病奈瑟菌	粘质沙雷菌	齿双歧杆菌	贝纳柯克斯体	伊氏放线菌
腐生葡萄球菌	大肠埃希菌	摩根摩根菌	迟钝真杆菌	汉氏巴尔通体	牛型放线菌
头状葡萄球菌	痢疾志贺菌	霍乱弧菌	产黑色素普雷沃菌	五日热巴通体	内氏放线菌
藤黄微球菌	福氏志贺菌	副溶血性弧菌	结核分枝杆菌	多杀巴氏菌	黏液放线菌
肺炎链球菌	鲍氏志贺菌	拟态弧菌	麻风分枝杆菌	白喉棒状杆菌	龋齿放线菌
化脓性链球菌	宋内志贺菌	河流弧菌	堪萨斯分枝杆菌	百日咳鲍特菌	星形诺卡菌
无乳链球菌	伤寒沙门菌	创伤弧菌	鸟-胞内分枝杆菌	嗜肺军团菌	巴西诺卡菌
缓症链球菌	副伤寒沙门菌	幽门螺杆菌	脓肿分枝杆菌	铜绿假单胞菌	鼻疽诺卡菌
草绿色链球菌	肖氏沙门菌	破伤风梭菌	龟分枝杆菌	空肠弯曲菌	
口腔链球菌	希氏沙门菌	产气荚膜梭菌	偶发分枝杆菌	胚胎弯曲菌	
唾液链球菌	肠炎沙门菌	肉毒梭菌	流感嗜血杆菌	嗜麦芽窄食单胞菌	

病毒

甲型流感病毒	鼻病毒	轮状病毒	发热伴血小板减少综合征病毒	人疱疹病毒 7 型	埃博拉病毒
乙型流感病毒	腺病毒	诺如病毒	克里米亚-刚果出血热病毒	人疱疹病毒 8 型	人类免疫缺陷病毒
副流感病毒	2019 新型冠状病毒	星状病毒	人疱疹病毒 1 型(HSV1)	流行性乙型脑炎病毒	人类嗜 T 细胞病毒
呼吸道合胞病毒	SARS 冠状病毒	甲型肝炎病毒	人疱疹病毒 2 型(HSV2)	登革病毒	狂犬病病毒
麻疹病毒	普通冠状病毒	乙型肝炎病毒	水痘－带状疱疹病毒(VZV)	森林脑炎病毒	人乳头瘤病毒
腮腺炎病毒	脊髓灰质炎病毒	丙型肝炎病毒	人疱疹病毒 4 型(EBV)	西尼罗病毒	B19 病毒
人偏肺病毒	柯萨奇病毒	丁型肝炎病毒	人疱疹病毒 5 型(HCMV)	汉坦病毒	博卡病毒
风疹病毒	肠道病毒	戊型肝炎病毒	人疱疹病毒 6 型	痘病毒	

真菌

絮状表皮癣菌	石膏样小孢子菌	卡氏枝孢霉	厌酷球孢子菌	热带念珠菌	烟曲霉
石膏样毛癣菌	秕糠马拉色菌	疣状瓶霉	皮炎芽生菌	光滑念珠菌	镰刀菌
红色毛癣菌	球形马拉色菌	甄氏外瓶霉	巴西副球孢子菌	近平滑念珠菌	毛霉
铁锈色小孢子菌	申克孢子丝菌	链格孢霉	马尔尼菲篮状菌	克柔念珠菌	耶氏肺孢子菌
犬小孢子菌	裴氏丰萨卡菌	荚膜组织胞浆菌	白色念珠菌	新型隐球菌	

寄生虫

恶性疟原虫	间日疟原虫	颚口线虫	棘阿米巴原虫	隐孢子虫	链状带绦虫
三日疟原虫	刚地弓形虫	贝尔蛔虫	溶组织内阿米巴	等孢子虫	
卵形疟原虫	管圆线虫	阿米巴原虫	环孢子虫	蓝氏贾第鞭毛虫	

支原体/衣原体/立克次体/螺旋体

肺炎支原体	解脲脲原体	恙虫病东方体	鹦鹉热衣原体	梅毒密螺旋体
人型支原体	普氏立克次体	沙眼衣原体	问号钩端螺旋体	伯道疏螺旋体
生殖支原体	斑疹伤寒立克次体	肺炎衣原体	回归热疏螺旋体	奋森疏螺旋体

注释：病原微生物宏基因组项目检测范围包括但不限于以上病原微生物。

质控信息

	总序列数	总碱基数	Q20	Q30	非人源占比
合格标准	>10,000,000	>750,000,000	>90%	>85%	
DNA	22,933,897	1,742,976,000	93.03%	90.10%	1.93%

注释：以上表格说明此次检测测序数据质量合格时，表明结果可信。

检测技术说明

病原微生物宏基因组检测项目基于第二代高通量测序技术，对特定样本中微生物群体的核酸序列进行检测分析，通过与数据库中微生物的核酸序列进行比对，从而鉴定样本中存在的可疑致病微生物。本检测通过高通量测序和智能算法分析，可检测 21388 种微生物，包括 11958 种细菌（其中包括 180 种分枝杆菌、251 种支原体/衣原体/立克次体/螺旋体）、7373 种病毒（其中包括 4414 种 RNA 病毒，2959 种 DNA 病毒）、1714 种真菌和 343 种寄生虫。

本检测项目在人源细胞含量为 10^5 个/mL 的样本中细菌的最低检出限为 100 CFU/mL，真菌的最低检出限为 500 CFU/mL，病毒的最低检出限为 500 拷贝/mL，低于检测下限时可能无法检出。当特定微生物未能检出时，不能排除受检者感染的可能性。为提高检出效果，建议在使用抗感染治疗前取样，并尽可能选取病灶样本，同时严格遵循样本保存运输的条件。

结果中所列的病原微生物以原核微生物、病毒、真菌、寄生虫进行分类，检测结果仅对该样本的本次检测负责。检测结果中提供的检出序列数可在一定程度上体现样本中微生物的相对丰度，不等同于样本中微生物的绝对含量。

主检	吴秋	审核	何媛	批准	汪静
主检实验室：长沙金域		网址：www.kingmed.com.cn<br>客服电话：4001-111-120			
如需了解更多请关注微信服务号“金域服务”		地址：长沙市高新技术开发区麓天路 28 号<br>邮编：410000			

人体皮肤常见正常菌落

（Allyson L.Byrd,et al，Nature Reviews Microbiology，2018）

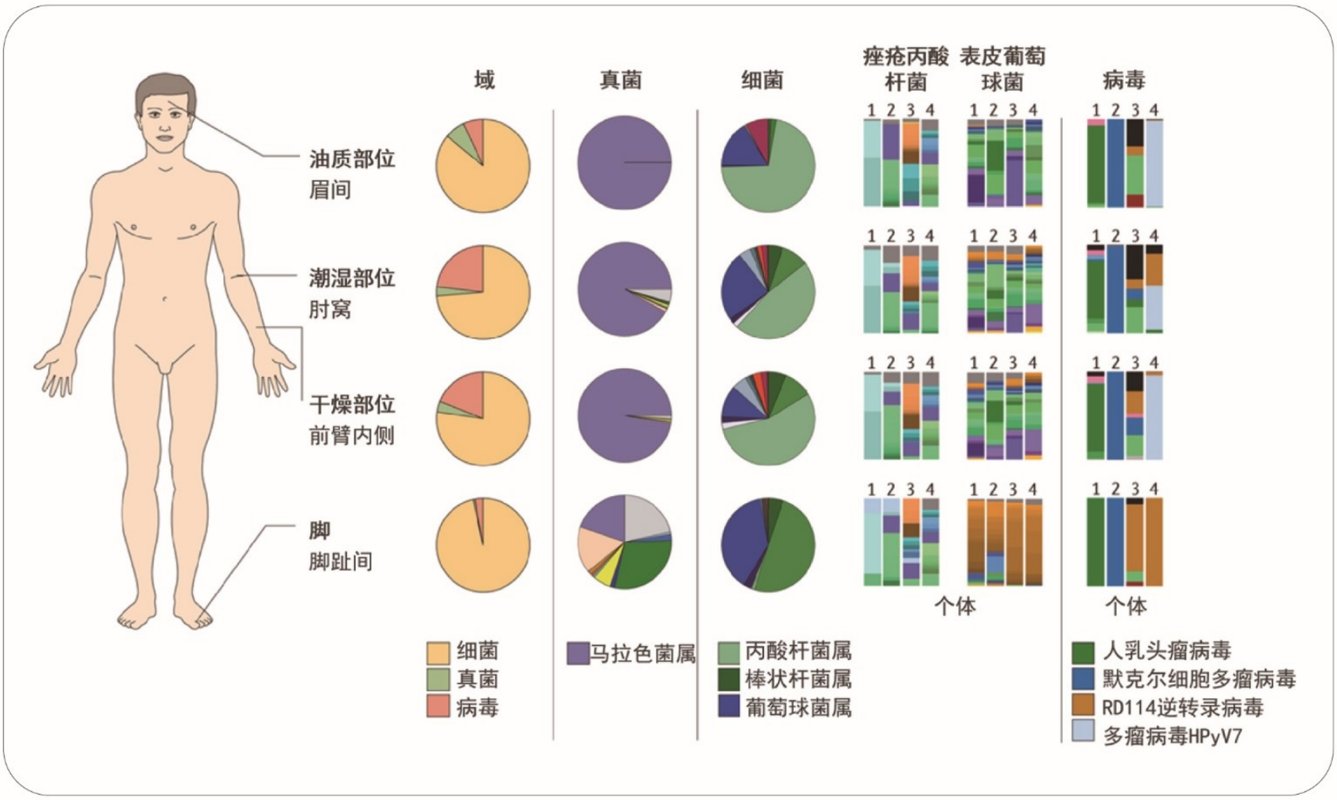

	手掌，前臂	鼻孔，肘窝，腹股沟折痕，腘窝	鼻翼，脸颊，眉间，外耳道，耳后，胸骨柄部，背部	脚趾间，脚趾甲，脚后跟
细菌	· 痤疮丙酸杆菌<br>· 结核硬脂酸棒状杆菌<br>· 轻型链球菌<br>· 口腔链球菌<br>· 假肺炎链球菌	· 结核硬脂酸棒状杆菌<br>· 人葡萄球菌<br>· 痤疮丙酸杆菌<br>· 表皮葡萄球菌<br>· 头葡萄球菌	· 痤疮丙酸杆菌<br>· 表皮葡萄球菌<br>· 结核硬脂酸棒状杆菌<br>· 头葡萄球菌	· 结核硬脂酸棒状杆菌<br>· 人葡萄球菌<br>· 沃氏葡萄球菌<br>· 表皮葡萄球菌<br>· 头葡萄球菌
真菌	· 限制性马拉色菌<br>· 球形马拉色菌<br>· 塔宾曲霉菌<br>· 近平滑念珠菌<br>· 合轴马拉色菌	· 球形马拉色菌<br>· 限制性马拉色菌<br>· 合轴马拉色菌<br>· 塔宾曲霉菌	· 限制性马拉色菌<br>· 球形马拉色菌<br>· 合轴马拉色菌	· 限制性马拉色菌<br>· 红毛癣菌<br>· 球形马拉色菌<br>· 须毛癣菌
病毒	· 传染性软状病毒<br>· 丙酸杆菌噬菌体<br>· 默克尔细胞多瘤病毒<br>· 多瘤病毒 HPyV7<br>· 人乳头瘤病毒（β）	· 传染性软状病毒<br>· 丙酸杆菌噬菌体<br>· 多瘤病毒 HPyV6<br>· 默克尔细胞多瘤病毒<br>· 多瘤病毒 HPyV7	· 丙酸杆菌噬菌体<br>· 传染性软状病毒<br>· 默克尔细胞多瘤病毒<br>· 多瘤病毒 HPyV6<br>· 人乳头瘤病毒（γ）	· 丙酸杆菌噬菌体<br>· 默克尔细胞多瘤病毒<br>· 人乳头瘤病毒（μ）<br>· 人乳头瘤病毒（β）

人体中枢神经系统感染常见病原

（Taojun He，et al，Curr Infect Dis Rep，2016）

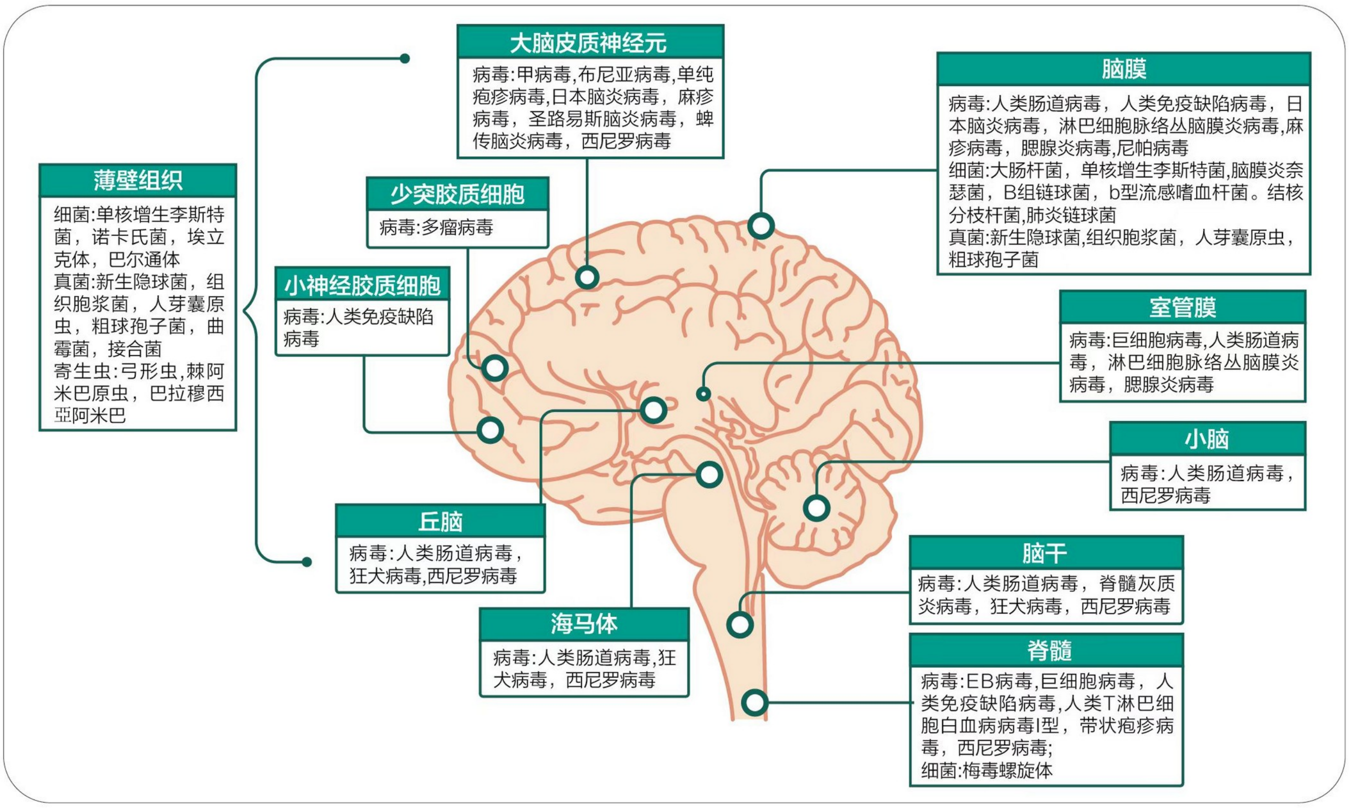

	原核微生物	真菌	病毒	寄生虫
脑炎	单核细胞增生性李斯特菌，诺卡氏菌，埃希氏菌属，巴尔通体属<br>人型芽孢杆菌，衣原体	新生隐球菌<br>曲霉菌属<br>酵母菌属	腺病毒，单纯疱疹病毒-1，单纯疱疹病毒-2，巨细胞病毒，人疱疹病毒-6，人疱疹病毒-7，细小病毒 B19 型，流感和副流感病毒，腮腺炎病毒，尼帕和亨德拉病毒，狂犬病病毒，西尼罗病毒，虫媒病毒，肠道病毒	弓形虫<br>变形虫
脑膜炎	大肠杆菌，单核细胞增生性李斯特菌，脑膜炎奈瑟菌，B 组链球菌，流感嗜血杆菌，结核分枝杆菌，肺炎链球菌，人型芽孢杆菌，衣原体	新生隐球菌	腺病毒，单纯疱疹病毒-1，单纯疱疹病毒-2，水痘-带状疱疹病毒，虫媒病毒，腮腺炎病毒，尼帕和亨德拉病毒，淋巴细胞脉络丛脑膜炎病毒，肠道病毒	
脊髓炎	结核分枝杆菌<br>梅毒螺旋体		水痘-带状疱疹病毒，人巨细胞病毒，人疱疹病毒 4 型，人类嗜 T 细胞病毒-1，人类嗜 T 细胞病毒-2，西尼罗病毒	

人体呼吸道常见正常菌落

（GB Huffnagle，et al，Mucosal Immunol，2018）

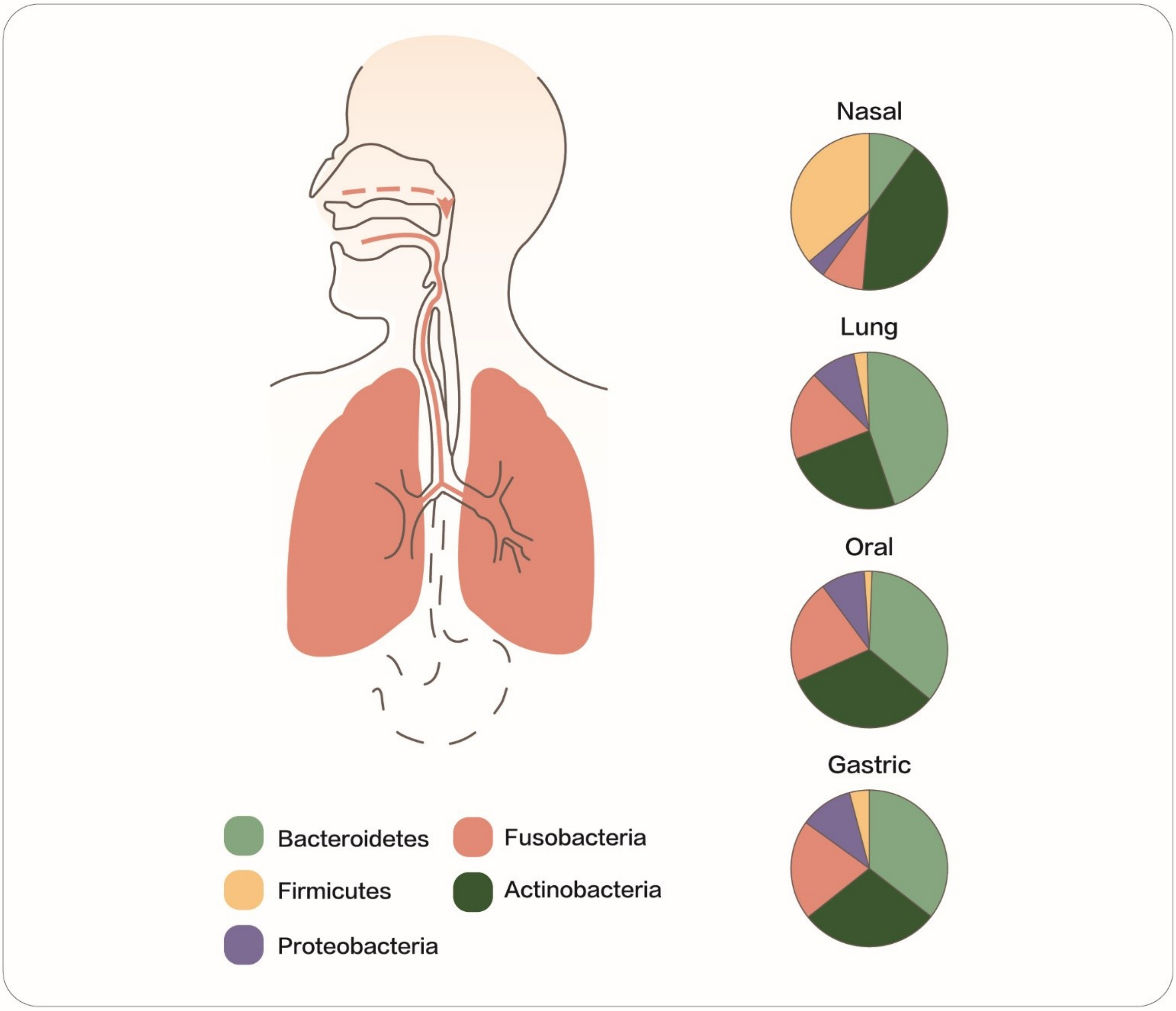

	葡萄球菌属	丙酸杆菌属	棒状杆菌属	莫拉菌属	链球菌属	嗜血杆菌属	罗氏菌属	奈瑟菌属	纤毛菌属	厌氧菌
鼻腔	√	√	√	√						
口腔			√		√	√	√	√	√	√
肺					√	√				

成为国内领先、国际一流的医学诊断信息整合服务提供商
To be a leading domestically and globally recognized provider
of integrated medical diagnostic information services
